# Is apathy a true trans-diagnostic construct? preliminary findings of the european study on apathy in schizophrenia

**DOI:** 10.1192/j.eurpsy.2021.160

**Published:** 2021-08-13

**Authors:** E. Fernandez-Egea

**Affiliations:** Department Of Psychiatry, University of Cambridge, Cambridge, United Kingdom

**Keywords:** motivation, negative symptoms, reward, apathy

## Abstract

Apathy is a quantitive reduction of goal-directed activity either in behavioural, cognitive, emotional or social dimension in comparison to the person’s previous level of functioning in these areas. Apathy is prevalent across many neurodegenerative, neurological, and psychiatric disorders. It represents the most common behavioural and psychological symptom in people with Alzheimer’s Disease and is often observed in Parkinson’s disease, vascular dementia, stroke, traumatic brain injury, amyotrophic lateral sclerosis/motor neuron disease, frontotemporal dementia, progressive supranuclear palsy, major depression, and schizophrenia. However, the definition and terminology employed to refer to apathy can vary in the context of different conditions and specialities and the diagnostic criteria have evolved. Additionally, the term apathy is employed to describe both a symptom and a syndrome. Indeed, little progress has been achieved in assessing the validity of the same construct across different disorders (eg. neurodegenerative disorders, schizophrenia or affective disorders). In 2018, a new version of the diagnostic criteria for apathy (DCA) in neuropsychiatric disorders was published. The validity of this new consensus has yet to be assessed among all relevant populations, including schizophrenia. Six European centres (Naples, Geneve, Nice, Rennes, Barcelona, Cambridge) aimed to test the prevalence of apathy, measured with the 2018 DCA, in patients diagnosed with schizophrenia. As a second aim, we focused on the relationship between DCA and other measures of apathy and negative symptoms in schizophrenia (BNSS and PANSS). In this talk, we will compare the preliminary findings of this pan-European study in schizophrenia patients with previous studies on neurodegenerative disorders.

**Disclosure:**

No significant relationships.

